# A Review of Risk Factors for Methamphetamine-Related Psychiatric Symptoms

**DOI:** 10.3389/fpsyt.2018.00603

**Published:** 2018-11-16

**Authors:** Xiangwen Chang, Yan Sun, Yang Zhang, Jiana Muhai, Lin Lu, Jie Shi

**Affiliations:** ^1^National Institute on Drug Dependence, Peking University, Beijing, China; ^2^Department of Pharmacology, School of Basic Medical Sciences, Peking University Health Science Center, Beijing, China; ^3^Department of Medical Experimental Science, School of Basic Medical Sciences, Peking University Health Science Center, Beijing, China; ^4^Peking University Sixth Hospital, Institute of Mental Health, Beijing, China; ^5^National Clinical Research Center for Mental Disorders and Key Laboratory of Mental Health, Ministry of Health, Peking University, Beijing, China; ^6^Beijing Key Laboratory on Drug Dependence Research, Beijing, China

**Keywords:** methamphetamine (MA), methamphetamine-related psychiatric symptoms (MAP), assessment instruments, risk factors, methamphetamine use related factors, socio-demographic variables and life events, genetic factors

## Abstract

The illegal use of methamphetamine (MA) is a growing public health concern globally and results in a series of negative effects. The prominent detrimental effect of MA use is MA-related psychiatric symptoms (MAP) and is observed at a much higher incidence compared to the general population. MAP often includes multiple dimensions of cognitive impairment and induces adverse consequences such as, violence and suicide. However, the assessment methods for MAP are not standardized. Hence, it is necessary to investigate factors that affect the progression of psychiatric symptoms in individuals who use MA. A review of published studies was performed by searching the following databases: PubMed, EMBASE, and PsycINFO from inception to 31 May, 2018. The search strategy included methamphetamine, dependence, psychiatric symptoms, and risk factor terms. We reviewed the different features of MAP and the various types of assessment instruments and summarized MAP risk factors from MA use-related factors, socio-demographic characteristics, life events, and genetic factors. We found that MAP was consistently and causally associated with MA use, particularly as it relates to the frequency and amount of MA use. Other MAP-related risk factors like life events and genetics were relatively inconsistent in their association with MAP. Hence, causal and longitudinal studies that focus on multilateral comparisons are required. This review provides high quality evidence for MAP risk factors and would be helpful for developing early prevention and treatment strategies for MAP.

## Introduction

Methamphetamine (MA) is a potent and addictive synthetic central nervous system stimulant. MA production and supply appears to be on the rise, particularly in East and South-East Asia 1. Based on the World Drug Report in 2018, around 34 million people are reported to be using an Amphetamine Type Stimulant (ATS) worldwide (1). Illegal MA use frequently leads to harmful physical and potentially debilitating mental health consequences (1, 2). Methamphetamine-related psychiatric symptoms (MAP) are the most common adverse consequences among MA users, with recent estimates of up to ~40% of MA users affected (3). The prevalence of psychiatric symptoms among MA users is 11 times higher compared to the general population (4). Several studies have reported that the prevalence of MAP in cohorts of MA abusers ranged between 15 and 23% in recreational or community settings, and up to 60% in MA addicts in treatment settings (5–8). MAP can exacerbate distress among individuals using MA and contribute to significant burden on health care services (9, 10). However, the risk factors for MAP beyond MA use are unknown with no effective indicators for early MAP warning.

MAP consists of transient psychiatric symptoms, persistent psychiatric syndromes, and long-lasting substance-induced psychiatric disorders (11). The transient MAP symptoms always manifest among people who use MA and gradually relieve after stopping using MA, last hours to months, and mainly include hallucinations and persecutory delusions (4, 12, 13). In contrast, persisting psychiatric symptoms generally involve irritability, anxiety, and depression (12). Compared to MA-associated psychosis symptoms which are acute, long-lasting MAP leads to a greater burden for individuals, family members, and even society (11, 14). In addition, MAP results in progressive social and occupational deterioration (15, 16), with several adverse effects including violence (17) and suicide (18). The likelihood of experiencing MAP is associated with a larger or higher frequency of MA dose (19, 20) and other MA-related factors. In co-morbidity psychiatric disorders, concurrent use of alcohol or other drugs and family history have also been associated with MAP risk (11). It is critical to understand to what extent MAP is attributable to MA use and identify additional risk factors for MAP. We reviewed comprehensively all MAP-related studies, particularly for studies that had persistent MAP. We then subsequently reviewed MAP related assessment and risk factors (based on classification, including: pharmacological, genetic, and environmental factors). We aimed to incorporate the latest reports and reviews in the field to provide high quality evidence for MAP risk factors.

## Methods

### Literature retrieval strategy

An extensive literature search was performed using PubMed, EMBASE, and PsycINFO databases from inception to 31 May, 2018. The search strategy included methamphetamine, psychiatric symptoms, and risk factors terms. Detailed search terms for methamphetamine include “MA dependence” or “amphetamine dependence,” and search terms for psychiatric symptoms contained “drug induced psychiatric symptoms,” “substance induced psychiatric symptoms,” and “psychiatric disorder,” and search terms for risk factors included “risk factors” and “vulnerability factors.”

### Inclusion criteria

To be considered for inclusion in this review, studies were required to have the following criteria:(1) human clinical study; (2) adults (>17 years) study; (3) studies where individuals using MA with current or lifetime MAP and were compared to those using MA without psychiatric symptoms; (4) MA was the primary drug; (5) studies which focus on MAP-related risk factors and describe the results clearly.

### Literature screening

We read the title and abstract of the papers for the initial screening. Then we did the second screening after reading the full text. Finally, we included the studies according to the inclusion criteria and extracted information from them. Two researchers independently completed the literature screening process. Any disagreements about study inclusion were resolved by discussion.

## Results

### Study characteristics

A total of 17 papers met the inclusion criteria and their characteristics were summarized in Table 1, which were sorted into four categories of MAP-related risk factors: MA use-related factors (10 studies), socio-demographic variables (10 studies), Life events(2 studies), Psychiatric co-morbidity(4 studies), and genetic factors (2 studies). Among them, there are 10 studies that focused on more than one category of risk factors.

**Table 1:** Studies identifying risk factors for methamphetamine-related psychiatric symptoms.

**Risk factors**	**Year**	**Author**	**Method**	**Country**	**Sample size**	**Measure of psychosis**
MAP related factors	2003	Chen et al.	Cross-sectional	Taiwan	445	DIGS-C MA-induced psychotic disorder
	2004	Lin et al.	Cross-sectional	Taiwan	325	FIGS-C
	2006	McKetin et al.	Cross-sectional	Australia	309	BPRS Current (past month)
	2009	Kalayasiri et al.	Cross-sectional	Thailand	96	MEQ MA-induced paranoia
	2009	Smith et al.	Cross-sectional	USA	205	Questions based on CIDI
	2010	McKetin et al.	Cross-sectional	Australia	75	Psychosis Screen Current (past year)
	2013	McKetin et al.	Cohort	Australia	278	BPRS Current (past month)
	2014	Ding et al.	Cross-sectional	China	189	MINI-plus MA-induced psychotic disorder
	2014	Kalayasiri et al.	Cross-sectional	Thailand	727	MEQ MA-induced paranoia
	2015	Hides et al.	Cross-sectional	Australia	198	PRISM-IV Lifetime and current psychotic disorder (substance induced or primary)
social factors	2004	Lin et al.	Cross-sectional	Taiwan	325	DIGS-C MA-induced psychotic disorder
	2004	Zweben et al.	Cross-sectional	USA	1016	MINI MA-induced psychotic disorder
	2007	Chen et al.	Cross-sectional	Taiwan	445	DIGS-C MA-induced psychotic disorder
	2008	Glasner-Edwards et al.	Cross-sectional	USA	526	MINI MA-induced psychotic disorder
	2009	Kalayasiri et al.	Cross-sectional	Thailand	727	MEQ MA-induced paranoia
	2013	McKetin et al.	Cohort	Australia	278	BPRS Current (past month)
	2014	Rognli et al.	Cohort (retrospective)	Sweden	1709	Hospitalization during 4- to 9-year follow-up
						Both primary and substance induced psychosis reported; data extracted for substance-induced psychosis outcome
	2014	Sulaiman et al.	Cross-sectional	Malaysia	292	MINI MA-induced psychotic disorder
	2014	Kalayasiri et al.	Cross-sectional	Thailand	727	MEQ MA-induced paranoia
Life events	2014	Rognli et al.	Cohort (retrospective)	Sweden	1709	Hospitalization during 4- to 9-year follow-up; both primary and substance induced psychosis reported; data extracted for substance-induced psychosis outcome
	2014	Ding et al.	Cross-sectional	China	189	MINI-plus MA-induced psychotic disorder
Psychiatric co-morbidity	2003	Chen et al.	Cross-sectional	Taiwan	445	DIGS-C MA-induced psychotic disorder
	2006	McKetin et al.	Cross-sectional	Australia	309	BPRS Current (past month)
	2016	McKetin et al.	Cross-sectional	Australia	278	Brief Psychiatric Rating Scale items
	2014	Sulaiman et al.	Cross-sectional	Malaysia	292	MINI MA-induced psychotic disorder
Genetic factors	2005	Chen et al.	Cross-sectional	Taiwan	445	DIGS-C MA-induced psychotic disorder
	2015	Hides et al.	Cross-sectional	Australia	198	PRISM-IV Lifetime and current psychotic disorder (substance induced or primary)

### Map assessments

Studies on MAP are sparse. They are mainly from existing data gathered from community hospitals or other research centers. Assessment of MAP is primarily based on clinician judgment. In targeted studies, investigators usually select appropriate research tools to assess certain type or types of mental disorders or symptoms and specific risk factors for which they are interested. Positive and Negative Syndrome Scale (PANSS) is used to assess MAP when clinical manifestations of MAP, especially transient MAP, are similar to schizophrenia (21–23). Beck Depression Inventory (BDI) is used to assess specific depression symptoms (24–26). Additionally, there are several other assessments used in MAP research, including the Beck Anxiety Inventory (BAI) (27, 28), Hamilton Depression Rating Scale (HDRS) (29, 30), and other scales (31–33). There is inconsistency in MAP definition when several assessments are compared (34–36). For example, the Mini International Neuropsychiatric Interview (MINI) could be used to assess lifetime MAP (16, 37, 38), while the list of psychiatric symptoms (Symptom Checklist 90, SCL-90) (39–41) could be used to assess recent MAP.

### The relationship between MA use and MAP

#### Duration of MA use

Earlier onset and longer duration (42, 43) are associated with increased risk of long-lasting MAP. Studies that associated these risk factors with MAP used lifetime measures of psychiatric disorders or symptoms (19, 44, 45). The severity of sustained MAP apparently related to earlier and longer exposure to MA, which reflects a “threshold” effect of stimulant use on the development of psychiatric symptoms (46).

#### MA use frequency and dose

Higher frequency and larger doses of MA use are closely associated with lifetime MAP (19, 20, 47). In a longitudinal prospective cohort study there was a five-fold increase in the likelihood of psychiatric symptoms during periods of MA use compared to sober periods (2). Frequent MA use had a relatively higher risk for longer-lasting MAP (44, 45, 48). Similarly, a higher dose of MA correlated with greater lifetime substance-induced psychotic disorders (19, 37) and psychiatric symptoms (19, 45, 49). These studies illustrate the dose-dependence relationship between MA use and the occurrence of psychiatric symptoms during periods of MA use (2, 50).

However, these relationships do not completely relate to all MA users. For example, there are certain subsets of individuals who do not appear to develop psychiatric symptoms with frequent MA use, while others suffer from chronic psychosis with limited exposure to the drug (51, 52).

#### Severity of MA dependence

Severity of MA dependence is closely associated with MAP. However, we found that the association was not consistent for all the studies evaluated. It is estimated that MA-dependent individuals were 2–3 times more likely to develop acute MAP symptoms compared to non-dependent individuals (19, 45, 53, 54). Nakama et al. found that the craving degree for MA use was correlated with the occurrence of persistent MAP (55). However, Hides et al. found that the severity of MA dependence was not associated with the risk of psychosis (6).

#### Route of MA use

There are several routes of MA administration, including intravenous injection and snorting. Individuals who use MA intravenously are more likely to develop persistent MAP (47, 56). However, some studies did not find any relationship between route of MA administration and MAP. Of these studies, there was one study that investigated recreational drug use and found a comparatively low rate of injecting use (57). Two of the studies reported current psychiatric symptoms (4, 57), with one reporting hospitalization for substance-induced psychiatric disorder (58) and the other describing lifetime substance-induced psychiatric disorder (37). In summary, additional studies should be conducted to validate the relationship between MAP and the route of MA administration.

#### Poly-drug use

Concurrent use of other non-stimulant drugs and alcohol is associated with an increased risk of persistent MAP (19, 59, 60), especially cannabis and alcohol use or dependence (2, 19, 45, 61). Ketamine may exacerbate persisting psychiatric symptoms of MA users, like anxiety or depression and anergia symptoms (62). To date, there is no agreement regarding the association between a lifetime history of other drug use and MAP. This may be due to ethnic differences or religion. There was one study conducted on cohorts from inpatient psychiatric and rehabilitation centers in a Muslim country (where consumption of alcohol is prohibited) and found that alcohol dependence was not associated with lifetime and current MAP (37).

### Socio-demographic risk factors

We found that several socio-demographic events had a correlation with MAP. However, certain controversies regarding the gender divergence of MAP has been noted. Several studies found that men were more prone to both transient and lifetime MAP (63–65), however other studies have demonstrated that transient MAP for both genders were similar (66–68). Regarding the age of onset, we found that young people with MA dependence were more vulnerable to acute and chronic MAP56 (69). Compared to non-MAP patients, patients with lifetime MAP were more likely to be single (65), unemployed (65), less educated (54, 65), and homeless (56, 58). Additional studies validating these findings need to be conducted.

### Life events

MA users with worse life experiences are likely to suffer from MAP, however there are inconsistencies with these findings. Studies have found that sexual abuse and risky sexual behaviors (56) of MA users are associated with developing persistent MAP (56), however several other studies have not found any association (58). One study investigated the impact of adverse childhood experiences (ACEs) on the development of transient MAP and found that individuals with three or more ACEs had a strikingly higher risk of MAP lifetime risk (44). One study documented that pre-morbid schizoid/schizotypal personality predisposes MA users to develop lifetime MAP (19). Several studies have supported the positive correlation of lifetime and current MAP and the diagnosis of antisocial personality and other behavior disorders (37, 45, 70).

### Psychiatric co-morbidity

There are inconsistencies regarding the association between concurrent psychosis and MAP. Pre-existing psychiatric symptoms in MA users are difficult to identify. Factors associated with susceptibility to schizophrenia appear to similarly predict MAP. McKetin et al. evaluated co-morbid affective and anxiety disorders among dependent methamphetamine users and found both transient and persistent psychiatric symptoms were associated with co-morbidity anxiety disorders and major depression (63). Additionally, MA users with a history of psychotic disorders (schizophrenia, schizoaffective, or bipolar disorder) were more likely to develop acute psychiatric symptoms (57, 65). Patients with severe symptoms of attention deficit hyperactivity disorder (ADHD) were more susceptible to transient MAP after agreeing to MA treatment (71), and MAP was more severe in patients with ADHD who underwent treatment that were previously consuming higher doses of MA (52).

### Family and genetic factors

Genetic and family factors also contribute to MAP. The MA users whose family members have a history of psychosis have a higher susceptibility to MAP. Relatives of persistent MAP patients had a higher risk of suffering from schizophrenia compared to relatives of transient MAP patients (72). Moreover, the degree of familial loading for schizophrenia could predict the onset and duration of MA psychosis (3). Several studies have demonstrated that first-degree relatives of individuals with MA psychosis were five times more likely to be schizophrenic (19, 72). McKetin et al. demonstrated that a family history of schizophrenia was associated with persistent MAP in MA users (70).

Currently, several classic substance dependence or psychiatric-related genes associated with MAP have been reported. Variations in *GRIN1* have been identified as a risk factor for schizophrenia and drug dependence, supporting the hypotheses of glutamatergic dysfunction in these disorders. The genetic variation of *GRIN1* provides a good example for the genetic association with MA dependence and its resultant psychosis (73). Ezaki et al. found that *5-HTTLPR* polymorphism were significantly associated with MAP, particularly among patients with prolonged MAP (74). In addition, polymorphisms in *DTNBP1* (75), *SOD2* (76), *COMT* (77, 78) may be associated with psychiatric disorders or symptoms caused by MA. A recent MAP GWAS analysis of Japanese cohorts appears to provide evidence that MAP shares genetic risk with schizophrenia, whereas no significant genome-wide SNP has been found (79).

## Discussion

We found that MAP was significantly associated with MA-related use factors, especially with the frequency and amount of MA use. Combined with the use of other addictive drugs, particularly novel synthetic drugs, like ketamine, may sharply increase the risk of MAP development. Although the number of studies regarding the association of life events and MAP were sparse, there was concordance with regards to adverse life events and MAP exacerbation. Substance dependence-related psychosis and psychiatric symptoms may usually affected by polygenic and genetic–environmental interactions. We did not cover all of these MAP risk factors. Other biological factors, such as serum brain-derived neurotrophic factor levels ≤ 1,251.0 pg/ml (24), disordered plasma immune factors (80), and HIV infection (81) of MA users have also been reported to be associated with MAP.

This review mainly focused on MAP rather than MA-associated psychosis that mostly represents acute symptoms. Although numerous studies and reviews have reported risk factors associating with MAP (3, 14, 82), several inconsistencies regarding these risk factors have made it difficult to conclusively determine their importance. The latest systematic review by Arunogiri et al. summarized the risk factors for methamphetamine associated psychosis from socio-demographic variables, methamphetamine use variables, other substance use, and family history (14), while other studies roughly described potential related risk factors (3, 82). Our review comprehensively outlines the risk factors for long-lasting psychiatric symptoms. In addition, to better understand MAP, we devoted greater attention to assessing MAP. The definition of MAP in this review is more extensive compared to simply defining it as psychosis. The risk factors we focused on were more comprehensive, and included genetic factors and ADHD.

However, these risk factors do not completely apply to all MA users. In addition to small or poorly representative sample cohorts, several confounding factors would affect the consistency of these findings. Firstly, poly-drug use is a common problem. The combined use of different drugs, like ketamine and cannabis, cause persistent psychiatric symptoms (62, 83), or cause transient intoxication states when cocaine is used (59). Secondly, only self-reported information from addicts was sometimes obtained. These reports do not help determine whether these addicts have MA exposure or not, or may not include other psychoactive substances including new (novel) synthetic drugs. In addition, most of the studies are cross-sectional, and the causal and prospective effects of MA use and pre-existing psychiatric symptoms could not be assessed. Hence these findings are not suitable for testing causality and predictive ability. In addition, the detrimental effects of MA use would be affected by purity and content of the drug itself. In addition, MAP assessments would be affected by recall or selection bias.

Psychosocial treatment for MA dependence is strongly evidence based and is the optimal first-line treatment strategy to reduce psychiatric symptoms in MA patients (3, 84). In addition, Cognitive-Behavioral Treatment (CBT) may be used to target multiple psychiatric disorders and symptoms, and emerging evidence supports the use of CBT to treat psychiatric symptoms associated with schizophrenia (85). For the treatment of acute MAP, guidance for clinical practice can be obtained from case studies. Numerous studies have reported the efficacy of antipsychotics including risperidone and aripiprazole for the management of acute MA-induced psychiatric symptoms (86). The treatment for long-lasting substance-induced psychiatric disorders should focus on MA abstinence to prevent future episodes of psychosis (3).

Early intervention can prevent MA users from progressing to disabling diseases like MA addiction and MAP (87). Future MAP-related studies should include individuals from community and treatment centers with a wide range of MA use histories to achieve more accurate and reliable study outcomes. Longitudinal, high-quality, and integrated studies which focus on risk factors for transient and long-lasting MAP are required. In addition, these studies should pay more attention to control confounding factors that affect the reliability of study findings.

## Author contributions

JS, LL, and YS conceptualized and organized the review study. XC, YZ, and JM performed the online study search and analysis. XC and YS wrote the manuscript. All authors edited and accepted the final version of the manuscript.

## Acknowledgments

This work was supported by the National Basic Research Program of China (No. 2015CB553503), the National Natural Science Foundation of China (No. U1402226, 81521063, 81601165, 31571099), the National Key Research and Development Program of China (No. 2017YFC0803608 and No. 2017YFC0803609). Beijing Municipal Science & Technology Commission (Z181100001518005 and Z161100002616006). And we appreciatively thank YS for helping us to proofread the language of this article.

### Conflict of interest statement

The authors declare that the research was conducted in the absence of any commercial or financial relationships that could be construed as a potential conflict of interest.
